# 

**Published:** 1884-12

**Authors:** 


### INDEX

TO THE

### INDEPENDENT PRACTITIONER.

### VOLUME V.

### ORIGINAL COMMUNICATIONS.

**A**bout Plastic Stoppings. C. E. F. . . . . . 435
Address to Graduates of the Philadelphia Dental College. S. H. Guilford. 169
Amalgam. S. C. G. Watkins . . . . . . 552
An Incident in Practice. C. E. Francis . . . . . . 142

**B**romides in Dentistry, The. R. M. Sanger . . . . . . 617
Bromide of Ethyl as an Anæsthetic. G. L. Curtis . . . . . . 297

**C**apillary Dentures. C. H. Land . . . . . . 243
Care and Treatment of Exposed Pulps, The. B. F. Luckey . . . . . . 430
Case of Fracture of the Inferior Jaw, A. T. B. Gunning . . . . . . 77
Case in Practice, A. C. H. Eccleston . . . . . . 557
Clinical Observations and Laboratory Experiments. C. M. Wright . . . . . . 613
Consideration of the Comparative Merits of Watts' Crystal Gold among Filling Materials, A. J. F. P. Hodson . . . . . . 607

**D**ental Education. B. Merrill Hopkinson . . . . . . 291
Dental Education Reply. Faculty Baltimore College Dental Surgery . . . . 358
Dental Education, Rejoinder. F. J. S. Gorgas . . . . . . 414
Dental Education, Rejoinder. B. M. Hopkinson . . . . . . 421
Dental Education, Rejoinder. Jas. H. Harris . . . . . . 425
Dental Education Once More. Faculty Baltimore College Dental Surgery. 491
Dental Hygiene. S. B. Palmer . . . . . . 237
Dental Mechanism. A. Retter . . . . . . 233
Day's Practice, A. N. S. Jenkins . . . . . . 1
D. D. S. in Europe, The. J. L. Tierney . . . . . . 241
Dental Notes in Different Parts of the World. G. H. Perine . . . . . . 71

**E**xperiments and Logic. Charles Mayr . . . . . . 193
Exposed Pulps. J. D. Patterson . . . . . . 487

Fermentation in the Human Mouth. W. D. Miller.....57, 113, 225, 281, 339

Herbst's New Method of Filling Teeth. W. D. Miller.................. 541
Herbst Method of Filling Teeth, The. C. F. W. Bodecker............. 597
Higher Dental Attainments. J. G. Palmer............................. 621

Incident of Practice. C. E. Francis................................. 253
In Union, Strength. Henry N. Dodge.................................. 301
Iodoform in Dental Surgery. C. F. W. Bodecker..................139, 177
Is Dental Work Expensive? D. R. Stubblefield........................ 250

Lecture on Pericementitis. Frank Abbott............................. 475

Microscopical Studies upon the Roots of Temporary Teeth. Frank Abbott 349
Multum in Parvo. J. Smith Dodge..................................... 141

New Method of Filling Teeth, A. C. A. Timme.......................... 27

Objectionable Clause, An. W. C. Starbuck............................ 678
On the Disposition of Time and Its Relation to Fees. A. W. Harlan.... 186
Over the Garden Wall. Is Dental Caries a Disease? C. M. Wright..... 135

Personal Recollections of a Dentist of the Early Days. L. W. Bristol... 662
Pivot Crown. F. E. Howard........................................... 368
Porcelain Facings for Carious Teeth. E. C. Moore.................... 433
Possibilities of Hereditary Transmissions of Oral Lesions, The. C. W. Starbuck.............................................................. 483
Professional Services and Professional Fees. E. T. Darby............ 653

Relation of Food to the Teeth. E. C. Kirk............................ 21
Remarks upon Diseases of the Antrum. Frank Abbott.................... 79
Reply to Some Views on the Putrefactive Theory of Decay, A. W. D. Miller............................................................... 15

Senile Decay. J. Smith Dodge, Jr.................................... 675
Sputum-Septicæmia. W. D. Miller..................................... 300
Suggestions for the Construction of Artificial Crowns and Bridges. J. H. Spaulding............................................................ 670
System as Applied to Instruments and Books. W. St. Geo. Elliott...... 65

Tin and Gold Combined as a Filling Material. W. D. Miller............ 403
To the Deans of the Dental Colleges. Adolf Petermann................ 427
Transplanting and Replanting of Teeth, The. A. H. Fuller............ 677
Treatment of Diseases of the Mouth. Julien W. Russell............... 408
Two Cases in Practice. J. L. Williams............................... 191

Use and Abuse of Gold as a Filling Material. A. N. Roussel........... 29

Visit to the Dentist, A. C. S. Stockton............................. 544

Wandering Supernumerary Tooth. E. S. Talbot......................... 558
Wisconsin Dental College, The. Adolf Petermann...................... 244
Woman; An Oration. N. W. Kingsley................................... 119

### CORRESPONDENCE.

Correction, A. T. H. Schaeffer........ 465

Dental Engines. F. W. Dolbeare........ 207

Error, An. J. Edward Line........ 209

From Rome. J. G. Van Marter........ 93

Gum Guillotine Forceps. L. D. Shepard........ 43

Incident, An. J. Smith Dodge........ 209
Iodoform. A. J. Reinhold........ 464

Marine Lint. E. D. Downs........ 464

Pre-historic Dental Diseases. Wm. H. Trueman........ 44
Pre-historic Teeth. W. D. Miller........ 40

The "M. D. S." C. S. Stockton........ 94

Warning to the Profession, A. A. W. Freeman........ 38

### REPORTS OF SOCIETIES.

American Dental Association........520, 560, 626, 693
American Dental Society of Europe........641, 689
American Medical Association........309, 380, 449, 507, 565, 623, 679

Central Dental Association of Northern New Jersey........458, 524, 699
Chicago Dental Society........85, 143, 319, 455, 516
Connecticut Valley Dental Society........ 32

Fifth District Dental Society of the State of New York........ 261

Illinois State Dental Society........374, 436, 511, 573

Meeting of the Faculties of the Dental Colleges of America........ 495
Mississippi Valley Dental Association........196, 254, 302

New Jersey State Dental Society........576, 631
New Orleans Odontological Society........ 148
New York Odontological Society........206, 258, 313, 461

Pennsylvania State Dental Society........568, 636, 683

Southern Dental Association........322, 369, 445, 528

Union Meeting of the Seventh and Eighth District Dental Societies of the State of New York........ 151

### EDITORIALS.

Abbott's Scissors........ 51
Advertisements........ 157
Afflictive........ 539
American Dental Association, The........ 465
Ancient Peruvians........ 50

Appeal, An .... 539
Archives of Dentistry .... 215
Assistant Needed .... 652
August Number Wanted .... 51
Author, The .... 648

Back Numbers Wanted .... 157
Bibliographical .... 102
Bogus Diplomas .... 266
Black's Micro-Organisms .... 537

Celluloid .... 103
Celluloid Handles for Instruments .... 103
Change, A .... 103
Commendatory .... 214
Contributors, To .... 391
Crowded Out .... 216
Current Number, The .... 467
Crowded Again .... 536

Dead Teeth in the Jaws .... 702
Deceased .... 648
Deferred .... 51, 332
Dental Education .... 331
Dental Education Again .... 209
Dental Societies .... 47

Earthy Phosphates, The .... 587
Editor's Special, An .... 646
Educational Problem—Victory, The .... 531
Electricity in Dental Practice .... 154

Fermentation in the Human Mouth .... 100
Filling Nerve Canals .... 262

Gross Misapprehension, A .... 647
Gum Guillotine Forceps *vs.* Gum Scissors .... 101

Half Year, The .... 392

Important Decision, An .... 393
Index Medicus .... 50
Item of Interest, An .... 327

Left Over .... 157
Legislation .... 329

Missing Number, The .... 648
Missing Numbers .... 468
Missouri Dental Journal, The .... 45
Model Report, A .... 707

National Dental Society, A ..... 97
Nausea ..... 215
New York State Dental Society Report ..... 332
Notice ..... 539

October ..... 585
Our Book Table ..... 158, 267, 332, 468, 649

Personal ..... 331
Personal—Our Colleague ..... 392
Pot *vs.* Kettle, The ..... 102
Protective Association ..... 538
Provoking Blunder, A ..... 589

Robinson's Fibrous Filling ..... 538

Shocking Occurrence, A ..... 536
Suggestive ..... 214

Text-Books ..... 588
Toothache ..... 216
Toothache Remedy ..... 266

Woman ..... 101

### OBITUARY.

Jarvis, Dr. O. A ..... 652

Long, Dr. W. H ..... 104

### ASKINGS AND ANSWERS.

Askings and Answers ..... 280, 338, 402, 474, 540, 596, 709

### CURRENT NEWS AND OPINION.

A. D. S. E ..... 336
American Dental Association ..... 401
American Medical Association ..... 276
American Monthly Microscopical Journal, The ..... 110
Anæsthetics from a Medico-Legal Point of View ..... 167
Analyses of Beef Peptonoids ..... 592
Another National Dental Society ..... 217
Assistant Needed ..... 652
Assistant Wanted ..... 595

Bogus Diplomas ..... 28

Can't Afford It........ 164
Carbolic Acid........ 55
Case of Œsophagotomy, A........ 276
Celluloid Disks........ 110
Central Dental Association of Northern New Jersey........ 222
Change of Date........ 337
Changes Produced in the Teeth by Syphilis........ 401
Change of Location........ 165
Cicero on Dentistry........ 277
College Commencements........ 218
Complimentary........ 166
Connecticut Valley Dental Association........ 279
Correction, A........ 167

Death of Cohnheim........ 595
Declined with Thanks........ 54
Dental College in France........ 166
Dental Education in Germany........ 594
Dental Legislation........ 52
Dental Meetings for May........ 275
Dental School in Berlin........ 707
Dental Society of the State of New York........ 275
Dental Society Meetings for April........ 223
Dentists Benevolent Association........400, 593
Dinner to Dr. Miller, The........ 394
Dissolution........ 110
Doctor Lord's "Sickles"........ 222

Error, An........ 221

Fifth and Sixth District Societies........ 595
Fine Chance, A........ 472
For 1884........ 111

German Graduates........ 51
Good Teeth and Good Intellect........ 54

Hay Fever........ 55
He Knew It........ 165

Illinois State Dental Society........ 273
In India........ 55
Iodoform in Surgery........ 223
It Pays Well........ 222

Journalistic Births and Deaths........ 223

Kansas State Dental Society........ 274

Legislation........ 220
Legal Decisions........ 661

**M**ad River Valley Dental Society.................................. 278
Massachusetts Dental Society.................................. 337
Meeting of Delegates.................................. 398
Michigan Dental Association.................................. 277
Michigan State Dental Society.................................. 163
Mississippi Valley Dental Society.................................. 165
Missouri.................................. 337
Mouth Mirrors.................................. 651
Muriate of Cocoaine.................................. 708

**N**aphthaline as a Disinfectant.................................. 108
National Association of Dental Examiners.................................. 400
New England Dental Society.................................. 595
New Jersey State Dental Society.................................. 399
New Local Anæsthetic.................................. 651
New Use for Electricity.................................. 166
New York Odontological Society.................................. 53
Northern Ohio Dental Association.................................. 279

**O**btuse.................................. 708
Odontological Society of Great Britain.................................. 167
Ohio State Dental Society, The.................................. 708

**P**ediatric Aphorisms.................................. 590
Pennsylvania State Dental Society.................................. 399
Perpetual Injunction.................................. 276
Personal..................................20, 278
Plaster Models.................................. 53
Please Remit.................................. 652
Popular Science News.................................. 4
Pre-historic Decay of the Teeth.................................. 473
Progress under Difficulties.................................. 682
Proper Medical Education.................................. 348

**R**egistering Vocal Sounds.................................. 221
Removal..................................111, 223
Removed.................................. 110
Resigned.................................. 54
Russian Teeth.................................. 109

**S**aratoga Meeting, The.................................. 473
Second District Dental Society of New York.................................. 278
Seventh and Eighth District Dental Societies of New York.................................. 595
Simple Operation for Facial Neuralgia, A.................................. 221
Sixth District Dental Society of New York..................................277, 337
Society Meetings for July.................................. 401
Southern Dental Association.................................. 279
Specific Disease in the Lower Animals.................................. 223

State of the Gums in Pregnancy........ 164
Supreme Court of Illinois........ 395

Teeth of the Future, The........ 105
Texas Dental Association........ 166
Therapeutic Agents for the Promotion of Osseous Development........ 106
Therapeutic Application of Nitrous Oxide Gas, The........ 272
Too Busy........ 271
Tooth Crown Patents, The........ 589
Tracheotomy for the Extraction of a Tooth from the Left Bronchus..... 55
Transactions of the New York State Dental Society........ 594
Transactions (N. Y.), for 1882–83........ 109

Units of Measurements........ 107
University of Buffalo........ 108
University of California........ 52
Unusual Case of Cleft Palate........ 559

Value of Artificial Respiration........ 111

Vulcanizing India Rubber........ 163

Western Miscellany........ 335
What Does It Mean?........

Zonular Cataract with Characteristic Teeth........ 106

### The Independent Practitioner—Vol. V.

### PUBLISHED BY DENTISTS FOR DENTISTS.

### Prospectus.

THIS JOURNAL is published by an association of professional men who have no ulterior objects other than to afford to the profession an independent, outspoken Journal, which shall be the exponent of higher aims and a better practice.

Having no connection with any school, clique, or advertising firm, it will be impartial in its judgment of professional matters, and its appeals for support are directed to those who believe that a liberal profession should have an independent literature.

While not neglecting practical details, it will pay especial attention to those oral diseases that have usually been under the care of the general practitioner, but which more properly belong to the specialty of dentistry.

Its pages will be open to any member of the profession who has anything of interest to communicate, and it invites a free expression of views on all subjects medical or dental.

As each of the gentlemen forming the association is practically a member of the editorial corps, it will have abundant opportunity for collecting all the latest professional news and intelligence.

It will have a body of regular contributors, selected from the very best writers in the profession, and will thus be certain of an abundance of interesting original matter.

It will pay especial attention to reports of the meetings of dental societies, and its aim will be to publish an epitome of all important discussions of professional matters.

It will contain original abstracts and translations of all that is of moment in the foreign journals.

Its profits, if any, will be devoted to its improvement by adding needed illustrations, paying for valuable original communications, and enlarging its size whenever this becomes advisable.

To the accomplishment of the work thus outlined (necessarily relying upon the hearty approval of an appreciative profession), each of the publishers of the INDEPENDENT PRACTITIONER has pledged his best efforts.

Editorial communications should be addressed to DR. W. C. BARRETT, No. 11 West Chippewa St., Buffalo, N. Y. Send all business letters to DR. WILLIAM CARR, 35 West Forty-Sixth Street, New York.

| | | |
|---|---|---|
| ABBOTT, FRANK....New York. | CARR, WILLIAM..New York. | JARVIE, WM. Jr..Brooklyn. |
| BARRETT, W. C.........Buffalo. | FRANCIS, C. E....New York. | NORTHROP, A. L.New York. |
| BODECKER, C. F. W. New York. | HILL, O. E..........Brooklyn. | PALMER, S. B......Syracuse. |

W. D. MILLER, Berlin, Germany.

